# *Citrobacter freundii* carrying *bla*_KPC-2_ and *bla*_NDM-1_: characterization by whole genome sequencing

**DOI:** 10.1038/srep30670

**Published:** 2016-07-28

**Authors:** Wenjing Wu, Björn Espedido, Yu Feng, Zhiyong Zong

**Affiliations:** 1 Center of Infectious Diseases, West China Hospital, Sichuan University, Chengdu, China; 2 School of Medicine, Ingham Institute for Applied Medical Research, Western Sydney University, Penrith, Australia

## Abstract

A carbapenem-resistant *Citrobacter freundii* strain WCHCF65 was recovered from hospital sewage and was characterized by genome sequencing and conjugation experiments. The strain carried nine genes encoding β-lactamases including two carbapenemase genes, *bla*_NDM-1_ and *bla*_KPC-2_. *bla*_NDM-1_ was carried on an IncX3 plasmid, which was identical to a plasmid found in a local *Escherichia coli*, suggesting interspecies horizontal transfer. *bla*_KPC-2_ was bracketed by two copies of insertion sequence IS*Kpn19*, which could form a composite transposon with the potential to mobilize *bla*_KPC-2_, on a new type of plasmid. The coexistence of *bla*_NDM-1_ and *bla*_KPC-2_ conferred higher levels of resistance to carbapenems compared with *bla*_NDM-1_ or *bla*_KPC-2_ alone. The coexistence of these carbapenemase genes, on two different plasmids, in one strain may allow new genetic platforms to be generated to mediate their spread.

Antimicrobial resistance of clinically significant bacteria is a global problem with carbapenem-resistant *Enterobacteriaceae* (CRE), in particular, emerging as a major threat for medical care. Carbapenem resistance among the *Enterobacteriaceae* is largely mediated by the production of carbapenemases, which are enzymes able to hydrolyse carbapenems and most other β-lactam antibiotics^1^. There are many types of carbapenemases, among which KPC and NDM were the two major groups produced in the *Enterobacteriaceae*^1^. *bla*_KPC_ and *bla*_NDM_ genes (encoding the KPC or NDM carbapenemases, respectively) are commonly found individually in CRE, but there have been several reports of isolates carrying both *bla*_KPC_ and *bla*_NDM_ worldwide^2^,^3^,^4^,^5^. We previously reported an *Enterobacter cloacae* carrying *bla*_KPC_ and *bla*_NDM_ from a blood culture of an ICU patient and demonstrated that the coexistence of both genes in that strain resulted in a slightly higher level of resistance to carbapenems^6^. During a study that specifically screened CRE from hospital sewage, we found a *Citrobacter freundii* isolate also carrying both *bla*_KPC_ and *bla*_NDM_. Here we report the genomic and plasmid characterization of the isolate.

## Results and Discussion

*C. freundii* WCHCF65 was recovered from the sewage input in West China Hospital, Chengdu, western China in January 2015. This isolate was resistant to ceftazidime (minimum inhibitory concentration [MIC], >1024 μg/mL), ciprofloxacin (8 μg/mL), imipenem (1024 μg/mL) and meropenem (512 μg/mL) but was susceptible to amikacin (4 μg/mL), colistin (1 μg/mL) and tigecycline (0.125 μg/mL). Among acquired carbapenemase-encoding genes screened, both *bla*_NDM_ and *bla*_KPC_ were detected in WCHCF65. *bla*_NDM-1_ and *bla*_KPC-2_ were identified by sequencing the complete β-lactamase gene sequences.

Both *bla*_NDM-1_ and *bla*_KPC-2_ were able to be transferred by conjugation, suggesting that they were carried by self-transmissible plasmids. Transconjugants obtained were found harbouring either *bla*_NDM-1_ or *bla*_KPC-2_, indicating that the two carbapenemase genes were carried by separate plasmids, designated pNDM1_CF65 and pKPC2_CF65, respectively. An *E. coli* J53 strain containing both plasmids was obtained by electroporation of a J53:pKPC2_CF65 transconjugant with purified pNDM1_CF65 DNA obtained from a respective transconjugant. A two- or four-fold increase in MIC was observed for imipenem and meropenem when comparing *E. coli* J53 with both *bla*_NDM-1_ and *bla*_KPC-2_ compared to J53 having either gene alone (Table 1). This confirmed our previous observation that the coexistence of *bla*_NDM-1_ and *bla*_KPC-2_ in a single strain could confer slightly elevated levels of carbapenem resistance^6^. Nonetheless, MICs of imipenem and meropenem against J53 containing both carbapenemase genes were still much lower than strain WCHCF65, suggesting that there were additional mechanisms mediating resistance to carbapenems. Previous studies have suggested that impermeability due to alterations of outer membrane porins could lead to resistance to carbapenems^7^,^8^.

Whole genome sequencing of *C. freundii* WCHCF65 generated 3,794,588 reads and 474,268,104 clean bases, from which 269 contigs ≥500 bp in length (N50, 72,950 bp) were assembled with a 51.9% GC content.

**Figure 1:**
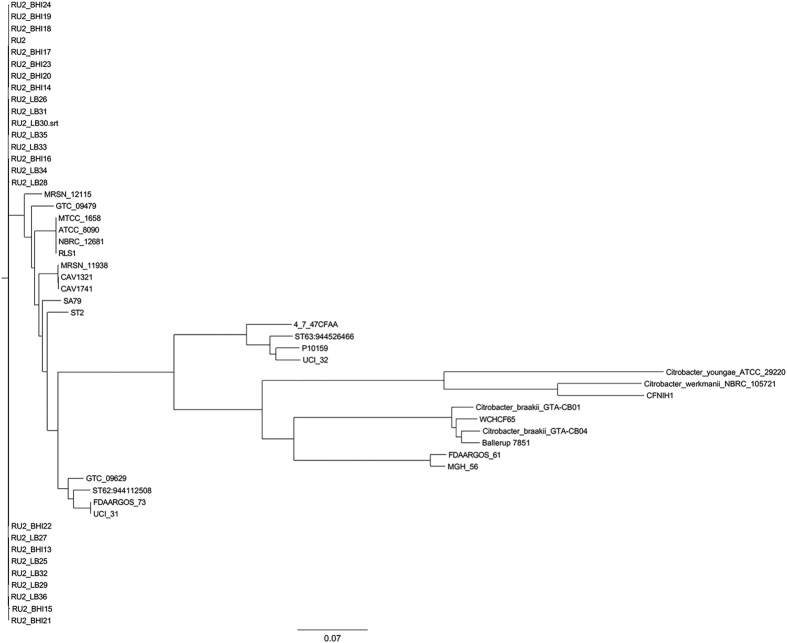
Phylogenetic tree of available *C. freundii* genomes. GenBank accession numbers of the strains are available at http://www.ncbi.nlm.nih.gov/genome/2850.

Strain WCHCF65 belonged to a new *C. freundii* sequence type: ST88. Among the other 48 *C. freundii* strains with whole genome sequences available in GenBank, strain WCHCF65 was closest to strain ballerup 7851/39 based on phylogenetic analysis. Interestingly, strain ballerup 7851/39 also belonged to a new ST (ST106) and was isolated from a female patient in 1939 in Denmark^9^ (Fig. 1). None of the seven alleles of ST88 were identical to those of ST106. The genome of WCHCF65 has the highest average nucleotide identity (ANI; 98.1%) with that of ballerup 7851/39, while the ANI between the genome of WCHCF65 and the remaining 47 *C. freundii* genomes varied from 90.22% to 92.91%.

Strain WCHCF65 had 16 known antimicrobial resistance genes, corresponding to its phenotype of resistance to carbapenems, ceftazidime and ciprofloxacin. In addition to *bla*_NDM-1_ and *bla*_KPC-2_, other β-lactamase genes were identified in strain WCHCF65 including *bla*_CTX-M-12_, *bla*_CTX-M-14_ and *bla*_SHV-12_ (encoding extended-spectrum β-lactamases [ESBLs]), *bla*_CMY-6_ and *bla*_CMY-137_ (encoding AmpC-type cephalosporinases), and *bla*_OXA-1_ (encoding a non-ESBL β-lactamase). Of note, this is the first report of *bla*_CMY-137_, which is chromosomally located and encodes a novel CMY enzyme, CMY-137 (assigned by the NCBI β-lactamase classification system: www.ncbi.nlm.nih.gov/pathogens/submit_beta_lactamase/). CMY-137 differs from its closest CMY enzyme, CMY-101, by a single amino acid substitution (Thr to Ser at position 195; position is assigned from the ATG start codon) but from CMY-6 by 19 amino acid substitutions (95% identity, 362/381 amino acids). As *bla*_CMY-137_ encoded a new CMY enzyme, it was cloned onto the pBC SK vector and was then introduced into *E. coli* DH5α. Transformants containing *bla*_CMY-137_ were resistant to ampicillin (MIC, >256 μg/mL), ceftazidime (32 μg/mL), cefotaxime (16 μg/mL) and cefoxitin (128 μg/mL) but were susceptible to piperacillin (16 μg/mL), piperacillin-tazobactam (1/4 μg/mL) and imipenem (1 μg/mL). The resistance spectrum conferred by CMY-137 was consistence with CMY-2^10^. The genetic context of *bla*_CMY-137_ was the same as other chromosome-based *bla*_CMY_ genes in *C. freundii* (*e.g.,* strain P10159; GenBank accession number CP012554) whereby *bla*_CMY-137_ and its *ampR* regulator gene were flanked upstream by genes encoding a fumarate reductase and a succinate dehydrogenase and downstream by a gene encoding an entericidin. The remaining seven resistance genes identified in strain WCHCF65 conferred resistance to aminoglycosides (*aac(6′)-Ib*-cr), chloramphenicol (*catB3*), fosfomycin (*fosA3*), macrolides (*mph*), quinolones (*qnrB10*), rifampicin (*aar3*) and sulphonamides (*sul1*).

pNDM1_CF65 is a 54-kb IncX3 plasmid that carries *bla*_SHV-12_ in addition to *bla*_NDM-1_ but no known acquired virulence factors. Interestingly, pNDM1_CF65 is identical to pNDM1_EC8 from an *E. coli* strain recovered in the same hospital in 2012^11^ and to pNDM-HF727 (GenBank accession number KF976405) from an *E. cloacae* strain isolated from Guangdong province, China in 2012^12^. The fact that the same plasmid has been found in different species of the *Enterobacteriaceae* at different locations suggests IncX3 plasmid-mediated horizontal transfer of *bla*_NDM-1_. It appears that IncX3 plasmids have emerged recently as a common vehicle mediating the transfer of *bla*_NDM-1_, particularly in China^11^ and *bla*_NDM-4_-like genes^13^.

**Figure 2:**
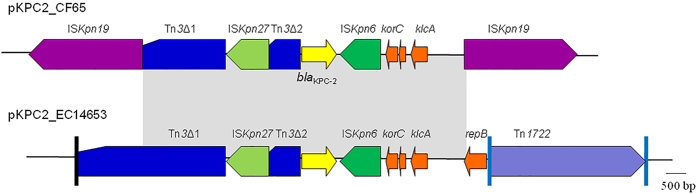
Genetic context of *bla*_KPC-2_ on pKPC2_CF65. Genetic context of *bla*_KPC-2_ on pKPC2_EC14653 (GenBank accession numbers KP868646), which was from a local *E. cloacae* clinical isolate, is shown for comparison. Regions seen on both plasmids are indicated by the grey shadings (nucleotide identity 99.8%). The inverted repeats of Tn*3* (black) and Tn*1722* (cyan) are indicated by vertical lines.

Plasmid pKPC2_CF65 was 40.9 kb in size and contained genes for conjugation and partitioning but genes encoding the replicon are yet to be determined. The backbone of pKPC2_CF65 had only 67% nucleotide identity (65% coverage) with the closest plasmid matches: p34399-43.500 kb (GenBank accession number CP010387) and p35734-109.753 kb (CP012163) and pCAV1669-34 (CP011648) from *E. cloacae*, pKPC_CAV1741 (CP011656) from *C. freundii*, and pCAV1374-34 (CP011629) from *Klebsiella oxytoca*. Both the PlasmidFinder tool and PCR-based replicon typing could not assign pKPC2_CF to a known incompatibility group. The above findings suggest that pKPC2_CF65 is a novel type of plasmid. On pKPC2_CF65, *bla*_KPC-2_ was the only known and complete antimicrobial resistance gene with no known acquired virulence factors being detected. Two copies of IS*Kpn19* flank *bla*_KPC-2_ on pKPC2_CF65 and potentially represent a composite transposon, which has not seen before. However, *bla*_KPC-2_ is seen in a similar immediate genetic context on pKPC2_EC14653, a plasmid from an *E. cloacae* isolate recovered in the same hospital^6^ (Fig. 2). On both plasmids, a Tn*3*-like transposon interrupted by the insertion of insertion sequence IS*Kpn27* can be found upstream of *bla*_KPC-2_ (Fig. 2). Downstream of *bla*_KPC-2_ there was a copy of IS*Kpn6* and several open reading frames including *korC* (function unknown) and *klcA* (encoding antirestriction protein) that are often seen with *bla*_KPC-2_ in other genetic contexts such as in pKPC2_EC14653. However, the *repB* gene (encoding a putative replication initiation protein), which was present on pKPC2_EC14653^6^, was truncated by the second copy of IS*Kpn19* in pKPC2_CF65. Given the similarity between the genetic contexts of *bla*_KPC-2_ between these two plasmids from the same hospital, it can be suggested that the insertion of two copies of IS*Kpn19* into a pKPC2_EC14653-like plasmid prior to the movement of *bla*_KPC-2_ via an IS*Kpn19*-mediated composite transposon or homologous recombination to a pKPC2_CF65 progenitor.

The coexistence of *bla*_NDM-1_ and *bla*_KPC-2_ has been previously identified in five enterobacterial isolates of different species (*i.e. C. freundii*, *E. cloacae*, *Enterobacter hormaechei* and *Klebsiella pneumoniae*)^2^,^3^,^4^,^5^,^6^. In all previous isolates and strain WCHCF65, the two carbapenemase genes were not located on a common plasmid. However, the coexistence of two or more plasmids in a single strain may allow new platforms for spreading carbapenemase genes to be generated. Of note, during this study, another *C. freundii* isolate (strain 112298) was reported to carry both *bla*_NDM-1_ and *bla*_KPC-2_, which was recovered from a patient in Guangzhou, southern China in 2013^5^. There was no obvious epidemiological link between strain 112298 and strain WCHCF65 that were recovered from these two hospitals (approximately 1,200 km apart in distance). pNDM_CF65 and the plasmid carrying *bla*_NDM-1_ in *C. freundii* strain 112298 were almost identical except a 3.5 kb inversion and 546 bp deletion in the latter plasmid. Both plasmids were also highly similar to other IncX3 plasmids from strains recovered in China^11^. In contrast, the plasmids carrying *bla*_KPC-2_ in the two *C. freundii* isolates had completely different backbones.

In conclusion, the study characterized a *C. freundii* isolate carrying both *bla*_KPC-2_ and *bla*_NDM-1_ that were carried by different plasmids. The coexistence of two carbapenemase genes in a single strain is of significance as it confers a higher level of resistance to carbapenems and may allow new platforms to be generated for mediating the further spread of the carbapenemase genes. Two copies of IS*Kpn19*, which bracketed *bla*_KPC-2_, could form a composite transposon, representing a new mechanism for mediating the mobilization of *bla*_KPC-2_.

## Material and Methods

### The isolate, *in vitro* susceptibility and screening for carbapenemase genes

Hospital sewage (1 mL) was obtained from the influx of the wastewater treatment plant in West China Hospital of Sichuan University, Chengdu, western China, in January 2015. The sewage was diluted 1:10 and an aliquot (100 μL) was spread onto a CHROMAgar Orientation (CHROMAgar, Paris, France) agar plate containing 2 μg/mL meropenem and then incubated at 35 °C overnight under aerobic conditions. Colonies were patched on CHROMAgar plates containing 2 μg/mL meropenem to ensure they were resistant to carbapenems and were then screened for acquired carbapenemase-encoding genes *bla*_GES_, *bla*_KPC_, *bla*_IMP_, *bla*_IMI_, *bla*_NDM_, *bla*_OXA-48_ and *bla*_VIM_ using PCR as described previously^14^,^15^,^16^,^17^. Amplicons containing the complete coding sequence of *bla*_NDM_^14^ or *bla*_KPC_^17^ were sequenced in both directions using an ABI 3730xl DNA Analyzer (Applied Biosystems, Foster City, CA, USA) at the Beijing Genomics Institute (Beijing, China).

Preliminary species identification was performed by partially sequencing the *gyrB* gene as described previously^18^. MICs of amikacin, ceftazidime, ciprofloxacin, colistin, imipenem, meropenem and tigecycline were determined using the microdilution broth method and breakpoints following recommendations of the Clinical Laboratory Standards Institute (CLSI)^19^. As no CLSI-defined breakpoints for tigecycline and colistin were available, those defined by the Food and Drug Administration (FDA) were used for tigecycline and those by the European Committee on Antimicrobial Susceptibility Testing (EUCAST)^20^ were used for colistin.

### Cloning of *bla*_CMY-137_

The complete coding sequence of *bla*_CMY-137_ was amplified with primers 65_N92-1EcoRI (AGAATTCAGTCGCTCAACAGAGGGAAA, the *EcoR*I restriction site was underlined; binding to *ampR*, upstream of *bla*_CMY-137_) and CMY137-dowEcoRI (AGAATTCTCGCCAGTGAAGTAGGCTTT; binding to a gene downstream of *bla*_CMY-137_). The amplicon was 2 kb in size and contained the 1,146-bp complete coding sequence of *bla*_CMY-137_. The amplicon was restricted using *EcoR*I (NEB, Ipswich, MA, USA) and then was ligated to *EcoR*I-restricted pBC SK vector (Agilent, Santa Clara, CA, USA) using T4 ligase (NEB). The ligated fragments were electroporated into *E. coli* DH5α and the *bla*_CMY-137_-containing transformants were selected on LB agar plates containing 100 μg/mL ampicillin. The presence of *bla*_CMY-137_ in transformants was confirmed by PCR and sequencing. MICs of ampicillin, ceftazidime, cefotaxime, cefoxitin, imipenem, piperacillin and piperacillin-tazobactam were determined using the broth microdilution method following recommendations of CLSI.

### Whole genome sequencing and analysis

Genomic DNA of strain WCHCF65 was prepared using QIAamp DNA Mini Kit (Qiagen, Hilden, Germany) and was subjected to paired-end (2 × 125 bp) whole genome sequencing with approximately 100× coverage using the HiSeq 2500 Sequencer (Illumina, San Diego, CA, USA) following the manufacturer’s protocol at the Beijing Genomics Institute. The Spades program^21^ was used to *de novo* assemble reads into contigs and annotation of the genomic sequence was carried out using the Prokka program^22^. ST was determined by using the genomic sequence to query the multi-locus sequence typing (MLST) database of *C. freundii* (http://pubmlst.org/cfreundii/) and the MLST tool from the Center for Genomic Epidemiology (http://genomicepidemiology.org/). Antimicrobial resistance genes were predicted using the ResFinder tool (80%, minimum threshold for identity; 60%, minimum coverage) from the Center for Genomic Epidemiology. To confirm the chromosomal location of *bla*_CMY-137_, the contig containing this gene was linked to a contig containing genes encoding ribosomal proteins by PCRs using primers designed based on available contig sequences and Sanger dideoxy sequencing. Acquired virulence factors were predicted using the VirulenceFinder tool (85%, minimum threshold for identity; 60%, minimum coverage) from the Center for Genomic Epidemiology to query the known virulence factors of *E. coli*.

Phylogenetic relatedness of WCHCF65 to the 48 *C. freundii* genomes available in GenBank (http://www.ncbi.nlm.nih.gov/genome/2850, accessed November 24, 2015) was investigated using the Harvest suite^23^. The ANI between the genome sequence of WCHCF65 and the 48 *C. freundii* genomes was calculated using the web-based JSpeciesWS tool^24^.

Plasmid replicons carried by WCHCF65 were determined using the PlasmidFinder tool (80%, minimum threshold for identity) from the Center for Genomic Epidemiology. The sequence of plasmids carrying *bla*_NDM-1_ or *bla*_KPC-2_ were completely circularised with gaps between contigs closed by Sanger dideoxy sequencing of amplicons from PCRs using primers designed based on available contig sequences. For pKPC2_CF65, PCR-based replicon typing was performed as described previously^25^.

### Plasmid manipulation experiments

Conjugation experiments of WCHCF65 were carried out in broth using azide-resistant *E. coli* strain J53 as the recipient; potential transconjugants were selected on LB agar plates containing 4 μg/mL meropenem and 150 μg/mL sodium azide. To obtain a J53 strain containing both *bla*_NDM_- and *bla*_KPC_-carrying plasmids (pNDM1_CF65 and pKPC2_CF65, respectively), plasmid DNA was prepared from J53 containing pNDM1_CF65 by alkaline lysis and was then electroporated into J53 containing pKPC2_CF65. Potential transformants were selected on LB agar plates with 250 μg/mL zeocin (Invitrogen, Waltham, MA, USA), a bleomycin antibiotic. As the recipient carried *bla*_KPC-2_ and was resistant to carbapenems, the use of carbapenems was therefore no longer able to distinguish the recipient and the transformant. Nonetheless, the recipient did not carry any bleomycin-resistant genes but the bleomycin-resistant gene *ble* was located downstream of *bla*_NDM-1_. Therefore, bleomycin could be used to screen the transformants. The presence of *bla*_NDM_ and *bla*_KPC_ in transconjugants and transformants were confirmed by PCR.

#### Nucleotide sequence accession number

Reads and the Whole Genome Shotgun Sequencing project of *C. freundii* WCHCF65 have been deposited into DDBJ/EMBL/GenBank under the accession numbers SRR2153225 and LIDR01000000, respectively. The sequence of pKPC2_CF65 has been deposited into DDBJ/EMBL/GenBank under accession number KU176944.

## Additional Information

**How to cite this article**: Wu, W. *et al*. *Citrobacter freundii* carrying *bla*_KPC-2_ and *bla*_NDM-1_: characterization by whole genome sequencing. *Sci. Rep.* **6**, 30670; doi: 10.1038/srep30670 (2016).

## Figures and Tables

**Table 1:** MICs (μg/mL) of carbapenems and ceftazidime against J53 containing one or both carbapenemase gene-carrying plasmids.

Strain	Ceftazidime	Imipenem	Meropenem
J53	0.5	0.03	0.25
J53: pNDM1_CF65	>1024	32	16
J53: pKPC2_CF65	64	32	8
J53: pNDM1_CF65: pKPC2_CF65	>1024	64	32
WCHCF65	>1024	1024	512
